# Learn, Engage, Act, Dedicate (LEAD): development and feasibility testing of a task-shifted intervention to improve alcohol use, depression and family engagement for fathers

**DOI:** 10.1186/s13033-022-00522-1

**Published:** 2022-03-04

**Authors:** Ali M. Giusto, David Ayuku, Eve S. Puffer

**Affiliations:** 1 Duke University Department of Psychology and Neuroscience, Duke University, 417 Chapel Drive, Durham, NC 27708 USA; 2 Duke Global Health Institute, 310 Trent Dr, Durham, NC 27710 USA; 3 Moi University, Academic Highway, Eldoret, Usain Gishu County, Kenya; 4 Present Address: Department of Psychiatry, Columbia University Medical Center/New York State Psychiatric Institute, 40 Haven Ave., #171, New York, NY 10032 USA

**Keywords:** Alcohol use, Fathering, Behavioral activation, Motivational interviewing, Low resource setting, Kenya

## Abstract

**Background:**

Men’s depression, alcohol use, and family problems commonly co-occur to create of cluster of mental health problems. Yet, few treatments exist to address these problems, especially in low and middle-income countries (LMICs). This paper describes the development and initial feasibility and acceptability of a novel task-shifted intervention to address this cluster of men’s mental health problems with a focus on engaging and retaining men in treatment.

**Methods:**

The intervention, Learn, Engage, Act, Dedicate (LEAD), is based in behavioral activation blended with motivational interviewing and was pilot tested in Kenya. To develop LEAD, we engaged in a community-engaged multi-step, collaborative process with local Kenyan stakeholders. LEAD was piloted with nine fathers reporting problem drinking. To assess initial feasibility and acceptability, recruitment and participation were tracked and descriptive statistics were generated given engagement of men was key for proof of concept. Semi-structured interviews were conducted with participants and analyzed using thematic content analysis.

**Results:**

The development process resulted in a weekly 5-session intervention rooted in behavioral activation, motivational interviewing, and masculinity discussion strategies. These approaches were combined and adapted to fit contextually salient constructs, such as the importance of the man as provider, and streamlined for lay providers. Feasibility and acceptability results were promising with high attendance, acceptability of delivery and intervention content, and perceived intervention helpfulness.

**Conclusion:**

Results describe an acceptable task-shifted treatment that may engage men in care and addresses a cluster of common mental health problems among men in ways that consider social determinants like masculinity. Findings set the stage for a larger trial.

*Trial registration* ISRCTN, ISRCTN130380278. Registered 7 October 2019—Retrospectively registered, http://www.isrctn.com/ISRCTN13038027

## Introduction

Problem drinking is a pervasive global mental health problem in high-income (HICs) and low and middle-income countries (LMICs) that accounts for 9.6% of disability adjusted life years [65]. Problem drinking is an umbrella term for various levels of harmful alcohol use and drinking detrimental to individual livelihoods and interpersonal functioning. In Kenya, problem drinking impacts men at a rate four times higher than women, with 21.3% of men and 5.1% of women reporting use [40]. Globally, problem drinking accounts for 7.6% of men’s deaths per year worldwide [18, 65], and is associated with a range of negative psychosocial, behavioral, and physical consequences [52, 53, 60]. Most common among these is depression [32]. Depression often co-occurs with drinking, so much so that drinking may be a diffuse indicator of mental health problems [24]. Further, among men, masculine norms that discourage help-seeking for depression has been hypothesized as a reason for men’s high rates of drinking worldwide (e.g., self-treatment; [19, 64]).

The consequences of men’s alcohol use and mental health problems often extend beyond the individual to impact families [29]. At the child-level, direct associations have been found between men’s drinking and youth substance abuse; social, emotional and behavioral problems; and poorer academic functioning [57]. Within the caregiver-child dyad, alcohol abuse influences the likelihood of child maltreatment, lack of paternal sensitivity, and decreased cognitive stimulation [23, 33]. In the couples’ relationship, intimate partner violence (IPV), marital conflict, and poor communication are all associated with men’s alcohol use globally [14, 35]. At the family level, pathways have been documented linking men’s drinking to a cascade of poor outcomes with associations between paternal drinking and child adjustment mediated by negative marital interactions and parenting [17, 56]. Given men’s alcohol use and depression often occur hand in hand with family problems, it is important to consider how to address this cluster of problems together.

Further, the nature, severity, and extent of this cluster are shaped in part by the context. These contexts, both ecological and cultural, need to be considered at the start of treatment development to ensure trialability and later scalability [13, 47]. One such factor is poverty, which impacts the severity of mental health consequences and the landscape of mental health care in an area. For instance, high rates of poverty have been associated with worsened alcohol use outcomes among men in LMICs as compared to those in HICs [18]. Further, fewer resources in LMICs, including Kenya, contribute to the large treatment gap (over 75% for depression alone) among individuals experiencing mental illness [66]. In Kenya, our formative qualitative work showed that men with problem drinking and their family members were unable to identify any formal treatment options in the area [44]. When asked about sources of assistance, men and family members exclusively noted informal help consisting of family and community members. As such, efforts to address the treatment gap must consider the paucity of mental health professionals, as well as the complexity of treatments. Task sharing—training non-professionals to provide treatment—and streamlining evidence-based practices for lay providers represent solutions [21] that have been proven effective for disorders including for substance use in Kenya and depression in India [43, 46].

Specific cultural factors, such as gender norms, can also influence multi-level consequences. Patriarchal norms in particular have been associated with men’s increased mental health problems and more severe consequences for men and their families [4]. In a Kenyan study, higher alcohol consumption was associated with more traditionally constructed gender among young men [37]. They have been shown to reinforce drinking to self-treat depression, interfere with help-seeking behaviors, and increase likelihood of family violence [15, 20, 69]. Given the importance of cultural norms, steps need to be taken to ensure mental health intervention content accounts for cultural values, language differences, and perceived drivers of illness. Formative work and collaborative processes with local stakeholders are two avenues for tailoring evidence-based practices in ways that will be viable and effective [5, 63].

In LMICs, effective, evidence-based interventions for problem drinking (e.g., Motivational Interviewing (MI; [41, 45]), depression (e.g., behavioral activation; PATEL), and family problems exist [6, 9, 26, 34, 48]. However, treatments addressing these commonly occurring problems simultaneously are scarce [16]. As such, there is a need for parsimonious treatments, conducive to LMIC delivery, that are tailored to engage men and target these interrelated problems together.

## Present study

The purpose of the current study was to develop a treatment to address men’s alcohol use, depressive symptoms, and co-occurring family problems in Kenya using a unique approach based in motivational interviewing (MI), behavioral activation (BA), and masculinity discussion strategies and examine the treatment’s initial feasibility and acceptability among fathers with problem drinking. We also sought to explicate a methodological process of collaborative treatment development that considers context, culture, and implementation factors at onset. Here, we first describe the process of intervention development and the resulting treatment (Part 1), followed by initial piloting methods, and resulting acceptability and feasibility findings (Part 2).

## Methods

### Part 1: intervention development

Here we describe multi-step, iterative process of treatment development and identification of broad treatment approaches foundational to the resulting intervention [63].

#### Development process methods

The first goal of the process was to identify any existing EBTs that target the cluster of outcomes (family, alcohol, mood) that were most likely to be feasible and acceptable in the Kenyan context. To this end, we began by analyzing previously collected qualitative data examining family interaction patterns associated with family functioning and child mental health (see [49, 50]). Qualitative results were based on focus group discussions with caregivers, youth, and mental health service providers in the area for a larger study on family functioning and youth mental health in the community. For this study, we focused on the findings related to fathers’ impact on the family system and child mental health. Findings showed two central themes: (1) the importance of gender-specific role fulfillment with the father as provider and (2) the pervasive burden of economic strain on men and family interactions. For fathers, this often led to an expectation to provide for family but an inability to do so. This inability was tied to feelings of purposelessness, idle time, disrespect from family, and conflict with partners. Drinking was often cited as a means to cope with associated difficult emotions, gain relief, and enjoy time with friends. Men’s drinking also seemed drive family conflict, with these fathers more likely to fight with family, act violently, not come home, or spend money on alcohol. Consequences of these behaviors included a lack of basic needs and, in some cases, the partner and child leaving. Data showed cycles of conflict, family problems, and depressive symptoms perpetuating drinking. These results informed our aim to treat depressive symptoms and alcohol use and improve family engagement by targeting these patterns.

Next, we aimed to identify evidence-based treatment (EBT) approaches that would form the foundation of the intervention. To do this, we conducted a systematic review of interventions targeting this cluster in LMICs, as well as a review of other evidence-based strategies targeting these outcomes globally [16]. The literature was then evaluated alongside qualitative results to assess the fit of treatment strategies with local needs, context, and amenability to lay provider use. Based on results from this process, we identified the therapeutic approaches that would be core to informing the intervention, described in the following section. Using these, we began developing the specific intervention. We first developed a theory of change to guide our process of combining, sequencing, and adapting the approaches for the context and for lay providers. The result was the first draft of the manual.

Using the initial manual draft, we then engaged in the next step—a process of collaborative development with a local team. The local team consisted of our Kenyan co-investigator (Author 2), individuals with mental health backgrounds (university students and community providers), local clinical supervisors, and lay counselors delivering the intervention. The process allowed for adaptations to occur at any point in development to optimize therapeutic strategies and their cultural relevance. After a full review by the two senior clinical psychologists from Kenya and the US, four individuals with undergraduate degrees in medical psychology from Moi University reviewed the manual, taking structured notes on feasibility, acceptability, understandability, and cultural relevance. Following revisions, we continued development by presenting the core intervention concepts, session content, and tools (i.e., worksheets, metaphors) to a group of nine Kenyan individuals with previous counseling experience (eight with a bachelors degree or higher in psychology; all with counseling experience). All concepts and materials were reviewed for acceptability, understandability, and cultural relevance. This led to another round of minor changes to metaphors, visualizations, and explanations.

Together, the above reviews led to iterative manual changes and a full revised second version with detailed session outlines. To pilot these, each session was role-played fully with two individuals with undergraduate degrees in medical psychology and previous counseling experiences to examine content flow and complexity, as well as language. During this process, key terms and concepts, such as “*urges*,” were identified, discussed, and translated for conceptual meaning and understandability. This step led to very specific adjustments and set the stage for full manual translation and the consistent translation of key terms. The full manual was then fully translated by one of the individuals involved in the role-play with experience translating mental health content. Each section was translated in order. After each section was translated, we evaluated comprehensibility and acceptability through discussion. To further revise the Kiswahili manual, the manual concepts were presented, discussed, and role played with lay counselor trainees to confirm conceptual understanding of translations and to ensure that the manual and materials were easy to use. Lastly, the intervention was tested with the first two participants in the pilot study, as described below; process data from session transcripts and selected supervision records were examined from these two cases for an initial assessment of understandability, feasibility, and acceptability prior to finalizing the manual.

#### Identified foundational treatment approaches

As described above, we combined our formative qualitative results, findings from our systematic review, and considerations related to lay provider and context delivery to identify evidence-based approaches to guide the development of the intervention. Three promising approaches emerged that matched needs, were parsimonious, had strong evidence, and were adaptable. These were: (1) Motivational Interviewing (MI); (2) Behavioral Activation (BA); and (3) Masculinity Discussion Strategies (MDS). MI and BA were identified as central mechanistic components from which to build a contextually feasible and culturally-congruent intervention. MI functions to increase intrinsic motivation to change and ambivalence about a behavior like drinking or treatment engagement [54]. BA aims to increase client engagement in value-aligned activities to increase positive reinforcement for behaviors inconsistent with drinking and/or depressive symptoms [22]. MDS are strategies used for expanding traditional conceptions of masculinity to include care and nurturance, as well as for exploring fathers’ models and beliefs about family [51].

MI has demonstrated efficacy reducing problem drinking and increasing mental health treatment engagement among men globally [67]. It is client-centered, increasing potential for adaptation with change in part predicated by the clients’ self-identification of values. We then chose to integrate BA given its efficacy reducing depression as well as multiple problems simultaneously, including substance use and depression in a low-resource United States settings [10]. It also uses a parsimonious approach [28], has shown widespread effectiveness across populations, contexts, and providers [7, 30]; and is anchored in personal values that are amenable to adaptation. Lastly, MDS was selected given its promise for improving family outcomes among men in LMICs, such as South Africa, as well as its focus on expanding ideas of ‘what it means to be a man’ given this emerged strongly in formative qualitative analysis [12].

Three existing treatment packages guided development. For MI, these were the mhGAP and the Screening, Brief intervention and Referral to Treatment (SBIRT) guidelines previously used in LMICs [1, 67]. For BA, this was Life Enhancement Treatment for Substance Use (LET’s ACT!); a flexible group treatment developed based on BA for depression to treat co-morbid depression and substance use disorder [11]. LET’s ACT targets the link between mood, use, and behavior by addressing goal-driven, non-drug forms of reinforcement [11]. For MDS, this was Program P: Manual for Engaging Men in Fatherhood, which compiles practices for engaging men in maternal and child health, caregiving, and violence prevention through a gender equity lens [51].

### Part 2: feasibility pilot trial

#### Setting and ethics review

Study activities were conducted in the Rift Valley Province of Kenya—a lower middle income country—in a peri-urban community surrounding the town of Eldoret. Eldoret is the largest town in the Province (population = 400,000), located on a main transportation route, and home to multiple ethnic groups. Historically, Eldoret and surrounding areas have experienced multiple periods of ethnic violence [61]. As in most LMIC settings, Kenya has very few mental health resources with two psychiatrists per 1,000,000 people [68]. All activities were done in collaboration with Moi Teaching and Referral Hospital (MTRH), the academic model providing access to healthcare (AMPATH), which is a consortium that included Moi University, MTRH, the Ministry of Health, and North American Universities. MTRH provides some psychiatric services, including limited inpatient and outpatient care. All procedures were approved by the Institutional Research and Ethics Committee at Duke University and MTRH; these included de-identifying all clinical data and storage on encrypted, password-protected devices.

#### Counselor recruitment

Three lay counselors were selected to deliver the treatment through a multi-stage process described elsewhere (redacted). First, community leaders identified ten men who they perceived to be role models in their communities and to have good listening and leadership skills. Men also were required to be fathers themselves and to have no prior counseling experience. We interviewed the group, invited six to a 10-day training, and made final selections based on training performance, including general counseling skills assessed with the ENhancing Common Therapeutic Factors Scale (ENACT; [27]), understanding of intervention theory and content, effective use of the manual, and receptivity to supervision.

#### Participant recruitment

We asked six community leaders and the six counselor trainees to refer fathers for treatment, with a target sample of ten participants. Participant eligibility was based on the following: (a) scoring between 8 and 20 on the alcohol use disorder identification test (AUDIT; [2]); responses were summed with higher scores indicating more problematic drinking patterns (α = 0.89) within the past 2 months and excluding those with possible dependence; (b) having a child between the ages of 8 and 17 years; and (c) having a partner willing to participate in assessment of men’s behavior to complement men’s self-report. At this proof-of-concept stage, we did not include depression symptom cut-off scores as part of eligibility as men often underreport or deny depression symptoms. Therefore, we choose to use drinking as the primary eligibility criteria given drinking is more observable and has been conceptualized as a diffuse indicator of mental health problems like depression. Throughout the trial, depression with the Patient Health Questionnaire, validated in Kenya [36, 59] was measured throughout.

#### Supervision

Supervision followed a tiered approach in which mental health professionals provide consultation to local supervisors who were trained to supervise lay counselors (redacted for review; [38]). For this study, three Kenyan supervisors were selected who had a bachelor’s degree in psychology. Local supervisors consulted with a US-based Masters-level clinical psychology doctoral candidate weekly to review cases and determine next steps. This consultant was in turn was overseen by a Kenyan clinical psychologist (Author 2) and a US clinical psychologist (Author 3) with weekly check-ins.

#### Measures

Below are measures and indicators used to assess initial feasibility.

*Recruitment tracking* All steps of the family recruitment process were documented throughout. From the beginning of the process, paper forms documenting recruiter and referee details for each participant were recorded in a database and used to track all referral sources; forms were completed by recruiters with the help of a project research assistant (RA). An RA in Kenya also recorded the number of men contacted, the number enrolled, the number eligible, and the number participating. This information was then entered into the database that was used to populate a data log with these numbers. Reasons for drop out or ineligibility were also recorded.

*Treatment attendance and exposure* Attendance logs were used to track and record participant attendance and attrition. Local supervisors completed logs based on post-session data forms completed by counselors and conversations with counselors. Scheduled sessions that were not attended with no 24 h notice given were considered to be a missed. Treatment exposure was measured by amount of time in each session and overall throughout the course of the treatment. Time exposed was documented using the length of session recordings and was cross-checked against post-session counselor notes on which the session time was recorded in order to account for any difficulties with recorder use. This was done for all participants.

*Qualitative interviews* Semi-structured interviews were conducted with each father. Participants were asked questions about the acceptability of the intervention, including content, materials and counselor, as well as about how helpful they perceived the intervention to be. All interviews lasted approximately one hour and were conducted individually in Kiswahili by trained research assistants not involved in intervention delivery. Interviews were audio-recorded, transcribed verbatim into English, and de-identified prior to analysis.

#### Analysis

Descriptive statistics were generated for attendance, attrition, and exposure. Qualitative interviews were analyzed using thematic analysis [62]. Two team members identified themes through close readings of transcripts, which were operationalized into a codebook. Two coders coded transcripts independently, double coding and reaching consensus on the first four. After reaching 80% agreement across codes on these transcripts, they were divided between coders, and thematic summaries were written for each to synthesize themes.

## Results

### Part 1: the intervention Learn, Engage, Act, Dedicate (LEAD)

The resulting intervention included MI, BA, and MDS strategies, which were integrated, streamlined, and sequenced in a way we expected would: maintain functional mechanisms of the foundational approaches, maximize cultural relevance, and facilitate lay provider use. We first present the overall approach with the theory of change followed by structure and session content.

#### Intervention approach: integrating and sequencing foundational components

**Fig. 1:**
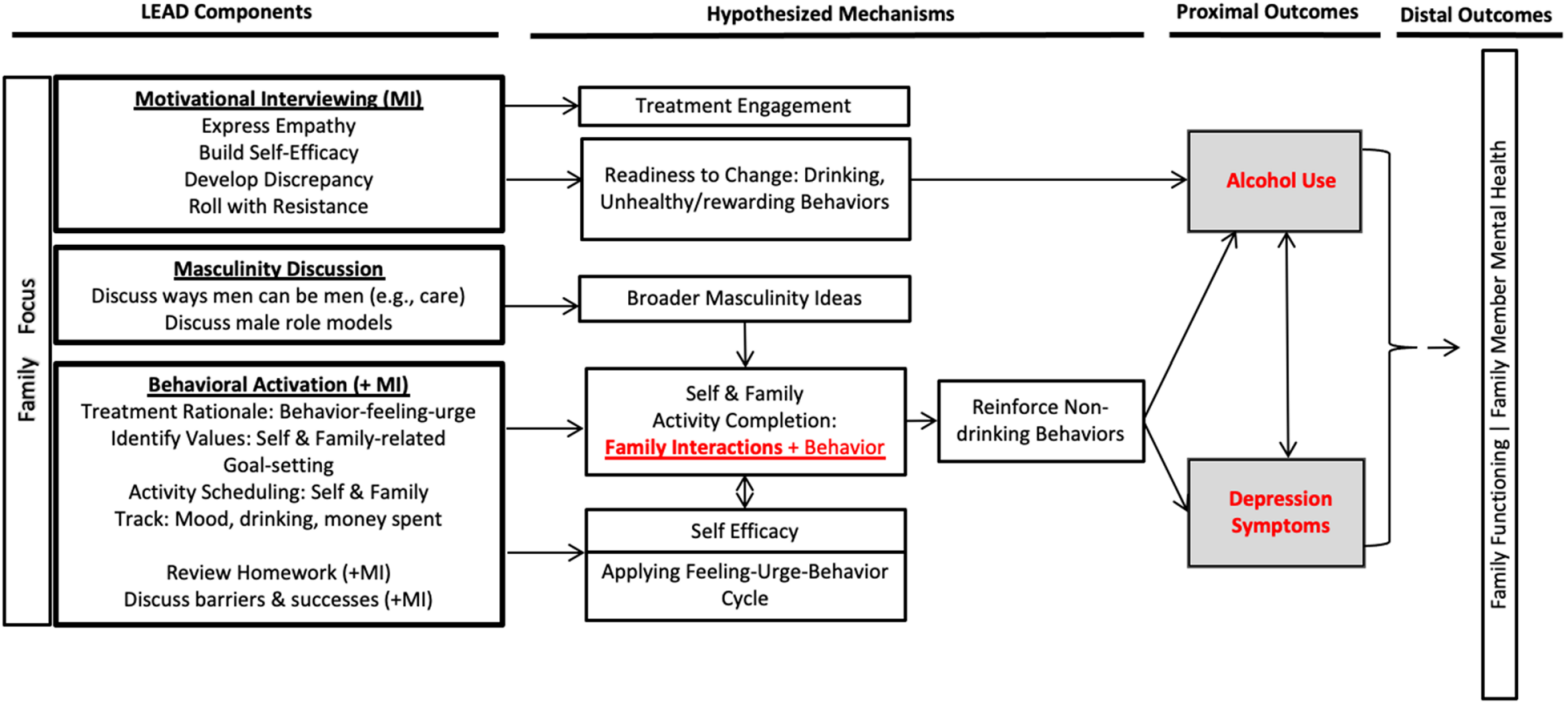
Learn, Engage, Act, Dedicate (LEAD) intervention theory of change. Red text signifies primary targets of intervention change

Figure 1 presents how the components were integrated in the context of the hypothesized theory of change. This presents how we expected the strategies to fit together to impact outcomes.

The MI-informed component is first introduced with two goals: (1) build readiness to change to drive reductions in drinking behaviors [39], (2) build participant motivation to engage in treatment, given that this is often a challenge for this population [55]. Additionally, counselors are trained to use MI principles such as empathy, partnership, and respect to guide interactions to enhance the therapeutic relationship.

BA is then the focus with MI integrated throughout. Fathers identify their values—one for self and one for family. They then identify non-drinking activities consistent with these values and schedule activities between sessions. The completion of both self and family focused activities is predicted to impact changes in both depression symptoms and alcohol use through increased family-directed and self-valued behaviors, hypothesized to increase reinforcement of healthy, valued behaviors. Aspects of MI are used to enhance client motivation to complete activities and homework as well as to build self-efficacy. Notably, asking the father to choose a family-based value is a more prescriptive strategy than typically suggested by BA or MI, yet its inclusion was consistent with culturally-specific data.

Consistent with BA, behaviors are tracked in detail. In this case, mood, drinking, and the amount spent on alcohol are monitored and reviewed each week to encourage behavior change. Additionally, counselors explain the rationale for treatment depicted in Fig. 3 to build participants’ insight related to the connections between emotions, urges and behavior. This is used to guide homework review to build men’s awareness of drinking and mood patterns. The goals of this are to (1) increase participant abilities to identify feelings that trigger drinking urges/low mood, to identify urges, and to choose alternative value-driven behaviors and (2) to improve participant ability to problem solve skills. Lastly, refusal skills are included. These were added after the first two pilot cases in which the influence of peers was cited as a key barrier to reducing use.

Aspects of MDS, often described as gender transformative strategies in the broader literature [12], are included to expand conceptions of hegemonic masculinity with the goals of (1) increasing motivation to engage in a variety of positive family-directed behaviors and (2) increasing men’s perception of the value of these new roles and activities. This is achieved by discussing men’s experiences with their own fathers—both positive and negative. As an example, a father identified that he enjoyed spending time with his own father yet feared when his father became angry with him for crying. The counselor guided the father to recognize that quality time and accepting emotional expression can be important aspects of the male caregiving role that can bring respect and positive interactions. Shifts in masculine beliefs were thought to shape family-focused values, drive family engagement motivation, and help motivate continued treatment engagement/help-seeking.

#### Intervention structure and session content

The intervention consisted of five 60–90 min sessions that occurred weekly, presented in Table 1. Prior to the first two cases, LEAD included only four sessions. Counselor and supervisor feedback prompted the addition of a session that provided more time for practice; Sessions 3 and 4 are therefore almost identical. The first session consists of MI-informed strategies, initial identification of values, and discussion of the treatment rationale. After a brief introduction, the counselor prompts the participant to relay what they, and then what their family, like and dislike about their drinking and mood. The counselor summarizes these and highlights the negative consequences. Next, importance of, and readiness to, change are assessed. After the scaling question, the client is asked why is he not a lower number to generate change talk. If readiness is below a three, this indicates that the participant likely is not prepared to engage in treatment, and the counselor engages in a procedure prescribed in SBIRT (i.e., discussing cons, showing concern, asking if they want information about drinking consequences, offer support). The counselor then asks if they are willing to discuss issues. If the participant says no, then the participant is provided referrals and information for reaching out when ready. If the participant says yes, then commitment is assessed; if they are committed, then they move to the next step. For those whose readiness is initially above a 3, the father is asked if he is ready to commit. If the participant affirms, they continue.

**Table 1:** LEAD intervention schedule

Session 1	Pros and cons of drinking: self and family Importance scaling question Readiness scaling question Confirm commitment program Vision for self Vision for family Value selection: self and family (value cards) Program invitation Path introduction and discussion (treatment rationale) Homework: track mood and drinking. notice positive activities
Session 2	Drinking and mental health assessment Homework and path review: drinking event and resisted drinking event Client’s relationship with father: positive and negative Client’s parents’ relationship: positive and negative Conceptions of masculinity: helpful and hurtful Value selection: self and family Activity scheduling Homework: complete activities. Track mood and drinking
Session 3	Drinking and mental health assessment Homework and path review: drinking event and resisted drinking event Refusal skills Value selection: self and family Activity scheduling Homework: complete activities. Track mood and drinking
Session 4	Drinking and mental health assessment Homework and path review: drinking event and resisted drinking event Refusal skills Value selection: self and family Activity scheduling Homework: complete activities. Track mood and drinking
Session 5	Drinking and mental health assessment Homework and path review: drinking event and resisted drinking event Value selection: self and family (client-directed) Activity scheduling (client-directed) Staying on the path: people and strategies to continue change Lessons learned: treatment review Graduate

**Fig. 2:**
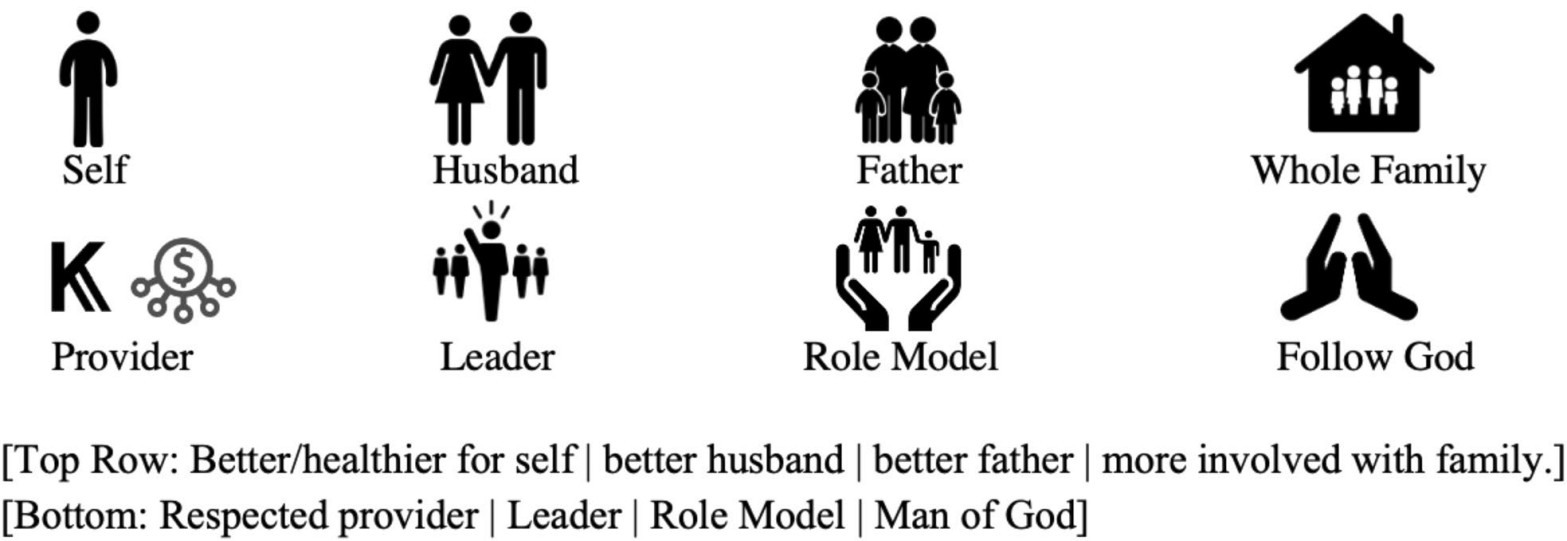
Value cards. Visual depictions of common values chosen to guide patient activity selection

**Fig. 3:**
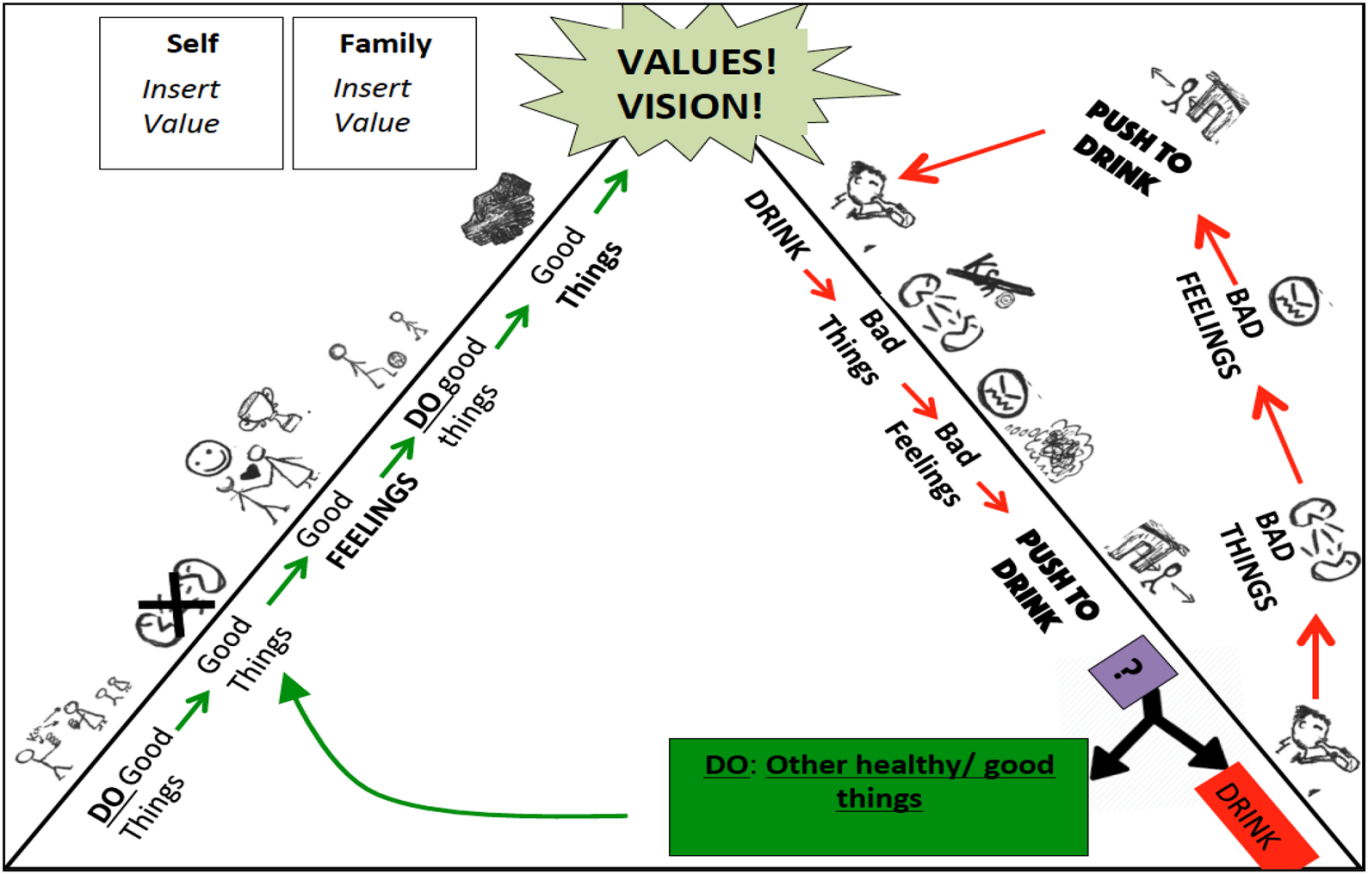
Treatment rationale—drinking and healthy paths: the counselor works sequentially from “bad things” on the pathway on the right with the red arrows (the “drinking” pathway). The counselor relays an example of a man progressing through this path. At each point, he prompts the client to guess what will happen next and then asks the client about their experiences. They then discuss where to break the cycle (green box) to walk a new healthy path (green arrows; left pathway) toward their identified values and vision

**Fig. 4:**
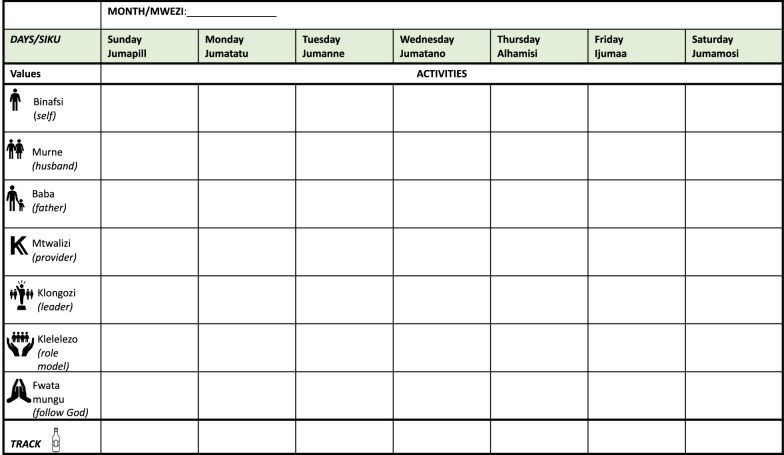
Activity scheduling sheet. The counselor helps the patient circle his value for the week that will guide activity selection. The counselor and patient write or mark the activity and time to the right of the values. The bottom line allows the patient to track his drinking with tally mark outside of session

After the participant states commitment, the session then focuses on identifying self and family-related values. The father is asked to share his vision for himself and his family using value cards—visual depictions of common values (e.g., being a good role model; Fig. 2). He is then asked how drinking and behaviors fit with his vision. After values are identified, the counselor uses a compass metaphor to explain how values can help guide life and treatment, which leads into a discussion of the treatment rationale using the paths depicted in Fig. 3. Here, the link between feelings, urges (i.e., *mskumo* in Kiswahili, translating to “push to drink”), and behaviors on the righthand path are emphasized with a focus on where to “leave the path” (forked arrow) and begin to engage in new non-drinking behaviors to reach values. This new sequence of behaviors and consequences is then depicted in the left pathway. Each path node is discussed using an example story to scaffold questions about the client’s experiences. At the end of Session 1, participants monitor pleasant non-drinking activities, mood and drinking patterns and are given notebooks with activity calendars (Fig. 4) and a picture of the path. Materials use visuals to depict information.

All subsequent sessions share the same basic schedule with some unique elements. The first step is a brief assessment of depression symptoms and drinking. Second, the counselor assesses participant readiness and confidence in change. If client readiness is above a 3, the counselor again asks why the participant is not a lower number than what he indicated. Third, homework is reviewed using MI-informed techniques. The counselor and participant then discuss a time the participant drank or engaged in behaviors tied to low mood and a time they resisted. Using prompts, the counselor summarizes triggers, positive behaviors, and affirms successes. Fourth, values for self and for family are discussed, chosen, and circled on the activities calendar; new values can be chosen each week. Fifth, the counselor guides the participant to brainstorm value-driven activities. Sixth, activities are collaboratively chosen, scheduled, and barriers to completion are problem solved. Lastly, homework is given to complete activities and track drinking and amount spent on alcohol.

Masculine Discussion Strategies (MDS) are used in Session 2. After assessment, the participant is asked to think of an object, smell, or feeling that connects them with their father and share that memory. They then discuss the father’s models of parenting and relationships through a series of semi-structured questions. Questions prompt the client to identify things they liked and did not like about those relationships, and what they would like to “take” or “leave” to enact in their relationships. Next, they discuss what it means to be a man, how those ideas might impact the way other men and fathers take care of children, and in what ways those ideas may have helped them or hurt them growing up. The participant is then prompted to discuss “*ways that we can ‘leave behind’ some of these ideas or look at them in a different way*,” as well as identify a role model who embodies these qualities. The counselor then summarizes responses to help clarify values and inform the next step of choosing values.

In sessions 3 and 4, unique content includes refusal skills introduced during homework review. Prior to their presentation, clients are asked if they would like to hear the skills. Some counselors role played these skills in session, but this was not required.

Session 5 includes a collaborative discussion of lessons learned and identification of others to whom fathers would like to relay program lessons. The father also identifies a person who can help keep them on their “healthy path.” Lastly, the counselor discusses specific areas of growth and presents the father with a certificate of completion.

### Part 2: pilot feasibility and acceptability

#### Father characteristics, attendance, and treatment exposure

A central question was whether recruitment, engagement, and treatment completion would be feasible. During recruitment, 27 fathers were listed by leaders and indicated a willingness to be contacted. Nineteen were able to be contacted, with one indicating he was not interested. The research team then visited the remaining 18 to assess eligibility and obtain consent. Thirteen fathers were consented and eligible. Of the five excluded, four were ineligible due to AUDIT scores indicating dependence (these individuals were referred to a higher level of care within MTRH), and one reported sobriety for over 2 months. Of the 13 eligible and consented, three did not follow through with scheduling requests to complete the first assessment, and one father who met criteria at baseline then reported no drinking for over 2 months during pre-treatment assessment.

The final sample included nine fathers (target 10). The mean age of participants was 39 (range: 48–30) with an average baseline AUDIT score of 16 (SD = 3.57) and an average PHQ-9 score of 7 (SD = 3.47). All were married except for one man who was living with their partner. The majority were from the Kalenjin tribe. Seven were casual workers, while the remaining three had permanent jobs. Most had completed primary school, with a few having secondary school education. The median monthly income of fathers was 1500 Kenyan Shillings (range: 6000–500). The average household size was 4.9 individuals (range: 6–3). Except one, all children were biological children of caregivers in the study; five of whom were female.

Attendance and treatment completion were high. Eight of the nine fathers completed all sessions. The other completed the first three sessions, after which he could not be reached by the counselor. Though not clear, it seemed that both he and his spouse were disinterested, potentially influenced by involvement in homebrewing, which complicates decisions to reduce drinking. Treatment completers had consistent attendance at weekly scheduled meetings (97.6%), with only one father missing a scheduled session, which was rescheduled successfully. Mean session length was 91 min (SD = 10.72), with an average total intervention exposure of 7.3 h.

#### Qualitative results

Overall, fathers shared positive feedback related to intervention content and helpfulness, the session format and materials, and counselor characteristics. All fathers reported enjoying the intervention content. When asked to recall and reflect upon the most helpful content of the intervention, many noted learning to understand pathways of unhealthy behaviors, discussing their vision for their lives, tracking their mood and behaviors, and learning skills to reduce drinking. All fathers described planning activities to avoid alcohol use, and some recounted specifically how this was helpful. For instance, one father reported, “Spending time with my children helped me avoid being out there with other people who could have engaged me in bad behaviors.” Most fathers specifically discussed learning about “urges,” with one 30-year old father with four children describing:

I came to learn that there’s this thing known as [an] urge…that triggers one to do thing[s]… then you [do it]. That will be like a vicious cycle…. [going] back to the same habit [drinking, isolating] because of bad thoughts, so it keeps going round and round. Therefore it is important when one experiences such an urge to just ignore it and refrain because by doing so one is able to accomplish a lot of positive things.

All fathers reported they perceived LEAD as acceptable and helpful. Most reported that LEAD helped them reduce their alcohol use, improve their mood, and improve interactions at home; they described feeling hopeful, keeping the peace in the home and bringing home and/or saving money. One father noted that it gave him the belief that he was capable of stopping drinking, which helped him reduce. Prior to treatment, a few fathers reported feeling uncertain and afraid of counseling but seeing changes in themselves and at home increased their comfort. Fathers also reported positive perceptions of the counselors, reporting that working with counselors who were not from their own community was acceptable. Many reported feeling comfortable discussing issues openly with their counselors. Some also described distinctly positive aspects of their relationships, expressing that they felt cared for and nurtured.

Almost all men also reported acceptability and utility of session materials, including the notebook and activity schedules. This was supported by their high rates of homework completion and remembering to bring their notebooks. One father did note difficulty record-keeping and completing at home activities, saying: “The one of record keeping [was difficult]. It was the only difficult thing to do because most of the time I would forget to note down [do homework]. I was given a book that I was to take back when it is fully recorded, [but] … I didn’t find time to do it or I would forget.” However, this same father also reported the following about finding it useful:

“I would feel good because that was a record of my things that I do on a daily basis. So I was happy that I was able to have a diary of my own. And I was glad because it was like a guideline to me because initially I would not list or plan my things, I was not orderly. But now it is helpful because whenever I open the book and see the list of what to do…I am able to plan well and see what to do first and so on.”

Though asked, this father did not elaborate about what could be different to help record-keeping.

Most fathers further reported that the intervention length was acceptable, though two fathers noted that they wished they could continue sessions longer. One initial concern was whether the sessions would last too long. However, none of the fathers raised this as a problem, and no sessions were ended early.

Given our small sample, we wanted to explore what factors may have contributed to attrition for the one case that did not complete treatment. His interview data revealed environmental, interpersonal, and internal elements that may have played a role. First, the participant noted living in close proximity to an “alcohol den”—a home that produces local brew. Second, his son was circumcised during this time period, creating a celebratory occasion for heavy drinking. Third, his interview suggested that his wife may have been ambivalent about treatment given that she may have been working in a home producing alcohol. Fourth, during treatment the participant expressed a preference for home sessions to avoid travel, which may have indicated low treatment motivation. Lastly, when asked directly, the participant reported that he did not continue attending sessions because he lacked time.

## Discussion

Through a systematic and collaborative approach to intervention development that considered implementation factors at the outset, this study resulted in a five-session task-shifted intervention targeting alcohol use, depression symptoms, and family problems for fathers in low-resource settings. LEAD is rooted in behavioral activation (BA) and motivational interviewing (MI) with strategies included to address issues of gender and bolster treatment engagement. LEAD demonstrated initial acceptability, feasibility, participant satisfaction with high rates of attendance with a difficult to engage population, and positive reports of intervention content, structure, implementation, and helpfulness. The resulting intervention, LEAD, begins to fill a gap in the literature by presenting a promising approach for addressing co-occurring problems tailored to men using a unique BA approach in LMICs where treatments are scarce.

This study adds initial support for the use of BA and MI as a blended treatment in low-resource settings [58], as well as the use of BA to address depression and substance use in LMICs—a unique and growing area of treatment research [31]. MI strategies have been recommended for use in LMICs [67], and BA strategies are also increasingly being employed in low-resourced settings in HICs [10] and LMICs, particularly for delivery by lay providers [46]. This study builds on this work by exploring the combination in Kenya and by weaving MI into the entire treatment [3]. Findings demonstrate the ability to design and deliver an intervention that takes this approach with lay providers in a low-resource context. As MI and BA apply to multiple mental health symptoms, this points to the possibility that such an approach may be feasible for other types conditions or populations.

Masculine discussion strategies (MDS) in individual treatment for men is another unique intervention element piloted here. This builds on use of MDS to target gender-based violence in group-formats [12], paternal engagement, and beliefs about caregiving [4]. In deciding whether and how to integrate this content, qualitative work and local input during development were critical. Formative work emphasized the impact of traditional masculine norms on rigid and highly gendered family expectations (e.g., man = provider), and the contribution of this rigidity to drinking and depressive symptoms. These results drove our decision to address these explicitly. In considering LEAD’s usefulness across contexts, it is likely that gender will be relevant. However, formative research on context-specific gender norms within the family will be important for adapting content to salient norms.

Given the well-documented difficulty of treatment retention of men and fathers [20, 42] and the intentional use of MI and MDS as engagement strategies, the attendance rates were encouraging. Results were consistent with literature from HICs demonstrating MI’s efficacy improving treatment adherence and attendance [8]. Further, the one case who did not complete LEAD provided insight into what barriers that could be assessed or addressed early in treatment, such as partner treatment ambivalence, proximity to alcohol, and initial motivation to engage in treatment.

While retention of treatment starters was high, there was a notable decrease from those listed by leaders and counselors as possible participants (n = 27) and those who expressed interest (n = 19) to those ultimately meeting criteria (n = 13) and initiating treatment*.* Such a decrease emphasizes the need to continue to consider sensitivity and specificity of recruitment and initial engagement efforts in this population. Self-identification recruitment approaches such as pamphlets or announcement for those interested in treatment or snowball sampling procedures may represent other promising community-based recruitment approaches. Respondent driven sampling represents a particularly promising recruitment approach for community-based work that has shown promise with substance using populations in South Africa [25].

Although preliminary results are promising, several limitations should be considered. First, the small sample size with little demographic variability and within a limited geographic area precluded us from learning about a broader range of environmental facilitators and barriers to treatment acceptability and feasibility; future work should include larger, more diverse samples. Second, it is possible that fathers who participated had a certain level of readiness to change, as we did not assess readiness among those who were eligible but did not participate. Formally assessing this during recruitment in future studies may provide a cut-off score for which men are likely to engage in and complete such an intervention. As such, more research is needed on the feasibility and acceptability of this intervention among men with lower levels of readiness or perhaps more barriers to participation. Third, we excluded men showing indicators of alcohol dependence, though the tested intervention could have benefits for this group as well, likely in conjunction with more intensive treatment. Lastly, fathers did not speak much about MDS during interviews, limiting data on its acceptability and feasibility; it would be a beneficial next step to explore men’s openness to MDS strategies for future treatment refinement.

### Lessons learned

We learned three main lessons that may be relevant for others adapting or developing interventions for culturally diverse populations or men. First, in tailoring treatment for men and designing strategies to promote their engagement, we identified treatment elements that were perceived as useful by men—a population that it often hard to engage and retain in mental health treatment, especially in a setting where treatment is scarce and unfamiliar. Second, we learned about the valuable aspects of the collaborative process of development and conclude that this increased the acceptability of the intervention. Third, we gained a better understanding of strategies for balancing EBTs and cultural-contextual fit.

*Tailoring treatment for men* From the outset, our team tried to consider the unique difficulties involved in engaging and retaining men in mental health treatment. With these considerations in mind, a few approaches emerged as relevant: (1) approaching men in an open, non-judgmental manner, which aligned with MI strategies that place choice with the patient; (2) using male counselors with shared patient characteristics of fatherhood; (3) addressing masculinity directly in treatment; and (4) fostering a strong therapeutic alliance.

*Strategies for collaborative intervention development* Although the extant empirical literature is key for treatment development, the importance of contextually-specific formative work and community collaboration should not be discounted [63]. Collaboration with in country partners and stakeholders guided a clearer understanding of evidence-based elements applicable to a new setting and selection of strategies at the start of development conducive to later implementation. Here, this included streamlining content for lay-providers, choosing approaches anchored in personal values, and creating materials for a range of literacy levels. Further, iterative, collaborative development allowed for tailoring throughout that led to culturally-relevant examples, content, and visualizations.

*Strategies for balancing evidence-based treatment and cultural-contextual fit* Lastly, this study adds to the evidence that EBTs and culturally relevant approaches are not mutually exclusive. Two processes helped us choose and employ empirically supported treatment strategies that were compatible with culture and context. First, at the initial stages of development, we tried to balance the exploration of the evidence alongside the in depth exploration of cultural-contextual factors to identify approaches with strong evidence *and* relevance. This was done through literature review, expert consultation, formative work, systematic mapping of concepts and evidence, and collaborative partnerships. Second, by creating the theory of change based on EBT functions alongside culturally relevant targets, we were able to intentionally sequence the specific evidence-based strategies to maintain core mechanisms and maximize relevance.

## Conclusion

In this study, we developed a mental health treatment (LEAD) relevant for men that targets co-occurring alcohol-use, depression symptoms, and family problems for use with lay providers in a low-resource setting. Results are very preliminary, but promising, supporting the feasibility and acceptability of the weekly five-session intervention combining MI, BA, and MDS. Participants exhibited high rates of attendance, reported high levels of satisfaction with treatment, and perceived the intervention to be helpful. Results set the stage for pursuing a detailed exploration of clinical efficacy and implementation strategies.

## Data Availability

Available upon request.

## Abbreviations

LMICS: Low and middle-income countries
LEAD: Learn, Engage, Act, Dedicate
MI: Motivational interviewing
BA: Behavioral activation
EBT: Evidence based treatment
MDS: Masculinity discussion strategies
SBIRT: Screening, Brief intervention and Referral to Treatment
LET’s ACT: Life Enhancement Treatment for Substance Use
MTRH: Moi Teaching and Referral Hospital
AMPATH: Academic model providing access to healthcare
ENACT: ENhancing Common Therapeutic Factors Scale
RA: Research assistant
